# Should all trainees “do research”?

**DOI:** 10.1186/s13244-025-01940-8

**Published:** 2025-04-04

**Authors:** Steve Halligan, Stuart Taylor

**Affiliations:** UCL Research Department of Imaging, University College London, London, UK

Dear Editor,

We enjoyed reading Klontzas et al which described trainees’ exposure to research [1]. They state that trainees need “…training on research methods, support for research during training, training on grant proposal writing, and manuscript reviewing,” concluding that “equipping radiologists in training with advanced research skills” will advance the specialty. On the face of it, these are very sensible and laudable aspirations that few would disagree with. Nevertheless, we believe they merit deeper consideration.

In the UK, the Royal College of Radiologists encourages trainees to engage in research, yet our own survey revealed the vast majority have no allocated time nor statistical support [2]. This provokes a “do it yourself” attitude, but a statistician rightly pointed out that a couple of lectures at medical school “doesn’t make you a statistician any more than attending a first aid course makes us into clinicians” [3]. Most medical research is poorly designed and executed [4], something that has been known for decades [5]. Radiomics is a good example from radiology, with most studies woefully underpowered [6], and without endorsement in patient management guidelines. Insisting that every trainee author a research study is likely to encourage more low-impact, low-quality research at a time when this should be discouraged. It was stated nearly 30 years ago that we actually need “less but better research” instead of more [5]. Do trainees really need, “training on grant proposal writing” [1]? External funding is extraordinarily difficult to obtain, even for highly experienced researchers. We never involve trainees when writing grant proposals because we are far too busy helping mid-career researchers do this.

Ultimately, demanding that all trainees acquire “advanced research skills” [1] cannot happen without adequate infrastructure. Even then, is this aspiration desirable? It is rather like demanding all radiologists need advanced mammographic or interventional skills. We have long rallied against the notion that every trainee should, “do research”. Our UK survey found that many trainees seek research experience not because of any burning desire to advance the field but to achieve promotion [2]. Our own trainees tell us repeatedly that they will happily abandon research immediately they achieve a permanent post. Only a small proportion ever aspire to become research leaders: It is far more efficient and sensible to concentrate our effort on these. Indeed, such individuals often actively seek out research training and mentorship, frequently at other institutions if expertise is unavailable locally; several European Radiological Societies offer research fellowships. The ESGAR, for example, also offers peer-to-peer mentorship where intermediate-grade academics teach generic skills to junior colleagues who have expressed an interest in research. While we hope this may enthuse individuals to pursue a research career, this is not the primary purpose of the program.

For the rest, training should focus on how to assess research quality because far more radiologists will read research papers than write them. It is far more important that trainees can recognize biased, poorly powered research, because patients may be harmed if incorrect findings are acted upon [5]. So, we would encourage a training environment where the majority are not “forced” to do research, thereby allowing scarce expertise and resource to focus on the few for whom it is a genuine passion.
